# A Novel Genospecies of *Borrelia burgdorferi* Sensu Lato Associated with Cricetid Rodents in Brazil

**DOI:** 10.3390/microorganisms10020204

**Published:** 2022-01-19

**Authors:** Bárbara C. Weck, Maria Carolina A. Serpa, Marcelo B. Labruna, Sebastián Muñoz-Leal

**Affiliations:** 1 Departamento de Medicina Veterinária Preventiva e Saúde Animal, Faculdade de Medicina Veterinária e Zootecnia, Universidade de São Paulo, São Paulo 05508-270, Brazil; vet.weck@gmail.com (B.C.W.); lilabiovet@gmail.com (M.C.A.S.); labruna@usp.br (M.B.L.); 2 Departamento de Ciencia Animal, Facultad de Ciencias Veterinarias, Universidad de Concepción, Av. Vicente Méndez 595, Casilla 537, Chillán 3780000, Chile

**Keywords:** *Oligoryzomys*, spirochetes, small mammals, Lyme disease

## Abstract

*Borrelia burgdorferi* sensu lato (Bbsl) spirochetes thrive in sylvatic transmission cycles infecting vertebrates and their ticks. Rodents and ticks of the genus *Ixodes* are important hosts of these spirochetes globally. Although evidence suggests that *Borrelia burgdorferi* sensu stricto does not exist in South America, genospecies of the group (Bbsl) can be found in this region but have been poorly characterized from a genetic viewpoint, and data on their ecoepidemiology are still incipient. Aiming to detect the natural foci of *Borrelia* in Brazil, we targeted small mammals inhabiting seven forests fragments during a period of three years (2015–2018). Organs (lung) from two *Oligoryzomys* rodents over a total of 382 sampled mammals were positive, and we performed a molecular characterization of 10 borrelial genes to achieve a robust analysis. Phylogenetic trees inferred from 16S rRNA, *flaB*, *ospC*, and seven MLST loci (*clpA*, *nifS*, *pepX*, *pyrG*, *recG*, *rlpB*, and *uvrA*) support the characterization of a novel genospecies of Bbsl that we herein name “*Candidatus* Borrelia paulista” Rp42. Remarkably, “*Ca*. B. paulista” is phylogenetically related to *Borrelia carolinensis*, a genospecies that infects *Ixodes* ticks and cricetid rodents in North America. A previous study performed in the same area identified *Ixodes schulzei* feeding on *Oligoryzomys* rodents. Although this tick species could be considered a probable host for this novel *Borrelia* sp., further research is needed to confirm this hypothesis.

## 1. Introduction

*Borrelia burgdorferi* sensu lato (Bbsl) are host-associated spirochetes that thrive in sylvatic transmission cycles infecting vertebrates and ticks [1]. The *Ixodes ricinus* complex of ticks are the main vectors of Bbsl in the northern hemisphere [1]. However, species of *Ixodes* in southern latitudes of the world also maintain *Borrelia* infections in nature [2,3]. These ticks acquire spirochetes after they feed and remain chronically infected, and after inoculating saliva into their hosts’ skin, they transmit the bacteria [4]. Importantly, Bbsl includes human-pathogenic spirochetes, and at least seven genospecies (i.e., *Borrelia afzelii*, *Borrelia bavarensis*, *B. burgdorferi* sensu stricto, *Borrelia garinii*, *Borrelia mayonii*, *Borrelia lusitaniae*, and *Borrelia spielmannii*) have been reported as the etiological agents of Lyme borreliosis [5,6]. Although Lyme borreliosis has yet to be proven in South America, serological and molecular evidence for a Lyme disease-like illness, named Baggio–Yoshinari syndrome, has been iteratively published in Brazil [7]; however, the evidence is currently considered inconsistent [8].

Rodents are important reservoirs of Bbsl in nature [1] and common hosts for ticks of the genus *Ixodes* as well [9]. For instance, in North America, cricetid (Cricetidae) rodents have been implicated as reservoirs of *B. burgdorferi* sensu stricto (s.s.), *Borrelia bissettiae*, *Borrelia californiensis*, and *Borrelia carolinensis* [4,10,11]. While ticks feed, Bbsl transit from the tick gut to the vertebrate milieu, and a plasmid encoded protein, OspC, allows for the infection of mammal hosts [12]. OspC favors the evasion of the host’s immunological system; therefore, depending on the host, strain-specific adaptations would account for a genetic variability of this loci among Bbsl [13].

Although robust evidence for *B. burgdorferi* s.s. does not exist in South America, genospecies of the group have been detected in rodent-associated ticks from Argentina [14], and Chile [15,16,17]. Moreover, in Brazil, a sequence of the flagellin encoding gene (*flaB*) that clusters phylogenetically within Bbsl, was retrieved from *Ixodes longiscutatus*, also a rodent-associated tick [18].

In an attempt to recognize vertebrate hosts of Bbsl in Brazil, we performed genetic screenings in organs collected from a large array of mammals inhabiting forests in three states of the country. Our results show the circulation of a novel *Borrelia* sp. phylogenetically related to *B. carolinensis*, a genospecies that infects *Ixodes* ticks and cricetid rodents in North America [10].

## 2. Materials and Methods

**Figure 1:**
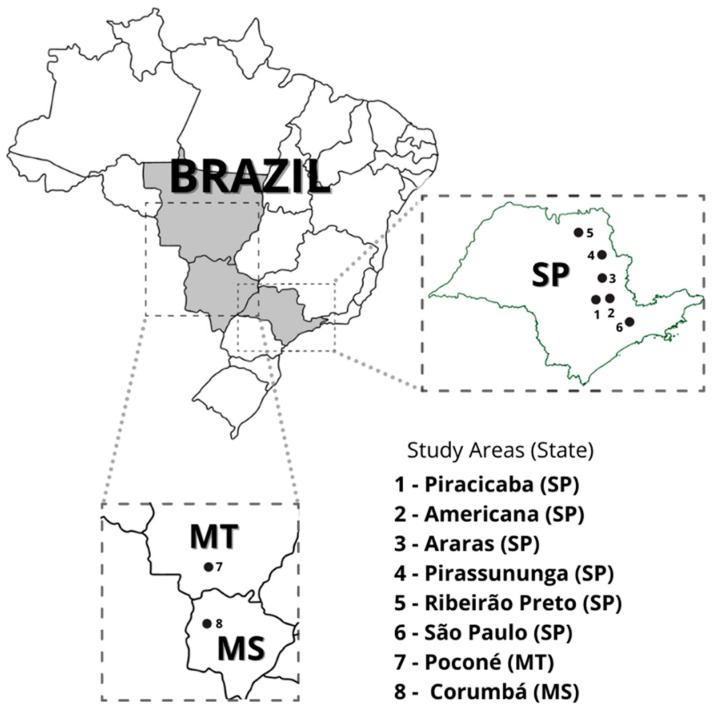
Areas in the states of São Paulo (SP), Mato Grosso do Sul (MS), and Mato Grosso (MT), where small mammals were captured.

Eight forest fragments were prospected: six of them located in the State of São Paulo, one located in the State of Mato Grosso do Sul, and one located in the state of Mato Grosso (Figure 1). Field work was performed during 2015–2018 in the dry (summer) and wet (winter) seasons with the aim to study the ecoepidemiological aspects of Brazilian spotted fever, as previously reported [19]. The protocols for animal handling are reported with detail in Serpa et al. (2021) [19]. Briefly, small mammals were captured with Tomahawk- and Sherman-like traps and anesthetized with an intramuscular injection of ketamine (100 mg/kg)–xylazine (10 mg/kg). At each locality, part of the captured animals was euthanized by increasing anesthetic doses, and necropsied to collect fragments of the spleen, liver, and lung, which were stored at −20 °C and transported to the laboratory. Only euthanized animals were evaluated in the present study. Animal carcasses were preserved in ethanol and identified based on taxonomic guides [20,21]. The above field protocol was authorized by IBAMA/ICMBio (SISBIO n. 43259-3), the São Paulo Forestry Institute (Cotec permit 260108-000.409/2015), and by the local Ethical Committee (Comissão de Ética no Uso de Animais, Faculdade de Medicina Veterinária e Zootecnia, FMVZ/USP), protocol numbers 5948070314, 6162060317, and 9531121015).

DNA extractions from organs were carried out using the DNeasy Blood and Tissue and Blood Kit (Qiagen, Chatsworth, CA), according to the manufacturer’s instructions. To verify the success of extraction, an initial PCR targeting the mammalian mitochondrial cytochrome b gene (*cytb*) was performed, as previously described [22]. Positive samples were then screened for *Borrelia* DNA with real-time PCR using genus-specific primers and a probe to amplify 148 base pair (bp) fragments of the *Borrelia* 16S rRNA gene [23]. *Borrelia*-positive samples were submitted to PCR protocols to obtain larger fragments of two borrelial genes: 16S rRNA [24] and *flaB* [25]. After sequencing those two loci and identifying that the detected *Borrelia* sp. belonged to the Lyme borreliosis group, we attempted to amplify the *opsC* [26], *clpA*, *clpX*, *pepX*, *pyrG*, *recG*, *nifS*, *rlpB*, and *uvrA* genes following a MLST scheme [27]. The primers and thermal conditions for *Borrelia* PCR are specified in the respective references. To confirm the identity of the *Borrelia*-positive animals, we sequenced the *cytb* amplicons.

PCR assays were performed in a total volume of 25 μL, using DreamTaq Green PCR Master Mix (Foster City, CA). *Borrelia venezuelensis* RMA01 [28] was employed as a positive control for the 16S rRNA and *flaB* genes. The DNA of “*Candidatus* Borrelia ibitipoquensis” [3] was used as a positive control for *ospC* and MLST PCR. Negative controls consisted of ultrapure water. Products were resolved in 1.5% agarose gels and amplicons with expected sizes, purified, and prepared for sequencing with the BigDye kit (Applied Biosystems, Foster, CA, USA). An ABI-PRISM 3500 Genetic Analyzer (Applied Biosystems, Foster, CA, USA) was employed for sequencing using the same primers for PCRs. The sequences obtained were subjected to BLASTn analyses to check their identities with the congeneric organisms available in GenBank [29].

The sequences generated in this study and the homologues retrieved from GenBank database were used to construct alignments for the 16S rRNA, *flaB*, *opsC*, and concatenated MLST genes using MAFFT [30]. Phylogenetic trees were inferred by Bayesian statistics using MrBayes [31], with four independent Markov chain runs for 1,000,000 metropolis-coupled MCMC generations, sampling a tree every 100th generation. Discounting burn-in of the first 25%, the remaining trees were used to calculate the Bayesian posterior probability. The general time reversible model was selected for all trees.

## 3. Results

A total of 382 mammals were euthanized: 7 species of marsupials, 18 rodents, 1 carnivore, and 1 cingulata. Samples of the liver, lung, and spleen were tested for each specimen (total: 1146 samples) (Table 1). Expected-sized amplicons for the *cytb* gene were obtained in all samples, thus confirming successful DNA extractions. Only the lungs of two *Oligoryzomys* sp. (Rodentia: Cricetidae) from Ribeirão Preto, São Paulo state (area 5), were positive for the *Borrelia* genus real-time PCR screening. Both animals were molecularly identified as *Oligoryzomys mattogrossae*, as we retrieved two equal *cytb* sequences that were 99.13% identical with *O. mattogrossae* from Brazil (KY952253, KY952255, KY952256, KY952258, and KY952259). A representative sequence of *cytb* generated in this study was deposited under GenBank accession number OL684651.

**Figure 2:**
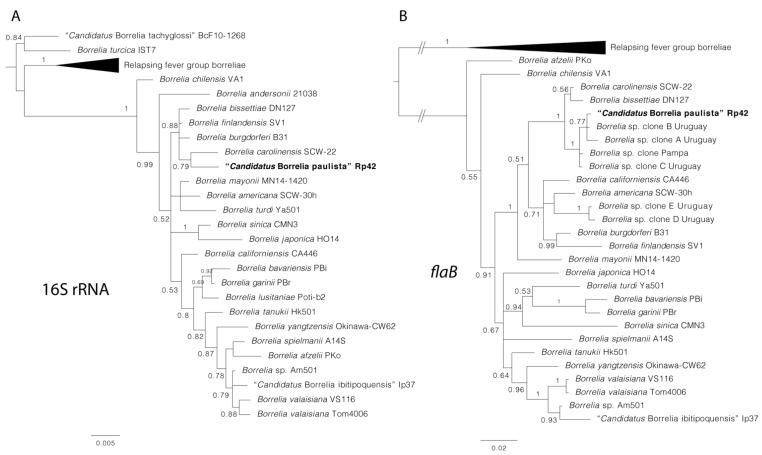
Phylogenies of a subset of *Borrelia* spp. using 16S rRNA (1419 bp) (**A**) and *flaB* (523 bp) (**B**) genes. Trees are drawn to scale with the scale bar indicating nucleotide substitutions per site. Values of Bayesian posterior probabilities ≥0.70 are indicated above or below each branch. The position of “*Candidatus* Borrelia paulista” Rp42 is highlighted in bold.

**Figure 3:**
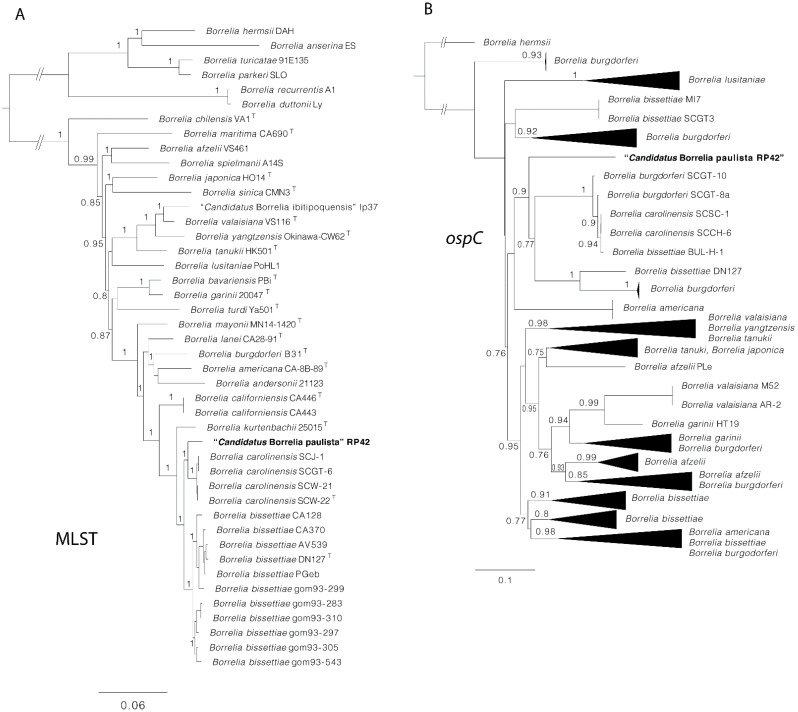
Phylogenies of *Borrelia* spp. using the MLST scheme (4132 bp) (**A**) and *ospC* (686 bp) gene (**B**). Trees are drawn to scale with the scale bar indicating nucleotide substitutions per site. Values of Bayesian posterior probabilities ≥0.70 are indicated above or below each branch. The position of “*Candidatus* Borrelia paulista” Rp42 is highlighted in bold.

We obtained fragments of the expected size for 16S rRNA, *flaB*, *ospC*, and seven of the eight MLST loci (*clpA*, *nifS*, *pepX*, *pyrG*, *recG*, *rplB*, and *uvrA*) in both positive *O. mattogrossae*. Pairwise comparisons proved that the *Borrelia* sequences from both rodents were identical with each other. Sequences of the 16S rDNA, *flaB*, *opsC*, and MLST genes were deposited in GenBank under accession numbers OL663845, OL631181-OL631189, and OL961816. Alleles 308-244-274-284-305-264-275 were assigned to *clpA*, *nifS*, *pepX*, *pyrG*, *recG*, *rplB*, and *uvrA*, respectively, and are available at http://pubmlst.org/borrelia/ (accessed on 15 January 2022). The phylogenetic analysis of borrelial 16S rDNA and concatenated MLST sequences indicate that the *Borrelia* sp. characterized from *O. mattogrossae* belongs to the Bbsl group and forms a monophyletic clade with *B. carolinensis*. On the other hand, *flaB* phylogeny points to a relatedness with South American genotypes detected in Uruguay and Brazil; *B. carolinensis* and *B. bissettiae* are also phylogenetically closely related with high support (Figure 2). Regarding *ospC*, the *O. mattogrossae*-derived sequence appears as an independent lineage and clusters within a group composed of *B. bissettiae* strains DN127 and BUL-H-1, *B. carolinensis* strains SCGT-8a and SCCH-6, and several *B. burgdorferi* s.s. strains from cricetid rodents or their ticks in the United States (Figure 3). The genetic and phyletic evidence retrieved in this study indicates that a novel genospecies of the genus was characterized, for which the name “*Candidatus* Borrelia paulista” Rp42 is proposed.

## 4. Discussion

In this study, we targeted eight forests fragments aiming to detect natural foci of *Borrelia* and found that *O. mattogrossae* harbors a novel genospecies of the Bbsl group. Rodents of the genus *Oligoryzomys* are ubiquitous along South American ecosystems [32], and their implications as reservoirs of *Borrelia* spp. are incipient. For instance, *Borrelia chilensis* was isolated from *Ixodes stilesi* ticks collected on *Oligoryzomys longicaudatus* in southern Chile [15]. Although the DNA of a Bbsl species was retrieved recently from this rodent species [17], its role as a reservoir of the spirochete is still obscure. Here, we detected “*Ca*. B. paulista” in organs of *O. mattogrossae*, implying that Bbsl would infect *Oligoryzomys* spp. Considering all of the sampled animals, a prevalence of 0.52% (2/382) for this spirochete seems to be low with compared with other ecosystems where Bbsl thrives [33,34]. It is well known that cricetid mice are common hosts for Bbsl in North America [35], and our results suggest that, in South America, rodents of this family maintain these spirochetes in enzootic cycles as well.

Eight areas of forest were targeted in our study, and positive animals were observed only in “area 5” (Ribeirão Preto). In the study of Serpa et al. [19], all *Oligoryzomys* specimens collected in “area 5” were determined as *Oligoryzomys nigripes* based on morphology and records of geographical distribution (data not shown). Herein, molecular analyses revealed that the two *Borrelia*-infected *Oligoryzomys* specimens belonged to the species *O. mattogrossae.* Given that we sequenced *cytb* from *Borrelia*-positive rodents only, that both species of rodents are morphologically similar, and that *O. nigripes* and *O. mattogrossae* might occur sympatrically [36,37], we cannot exclude that the two species were present in the same area. For this reason, we mention them in Table 1 as *Oligoryzomys* spp.

In a previous study performed in “area 5”, some *Oligoryzomys* specimens were infested by nymphs and larvae of *Amblyomma dubitatum* and *Ixodes schulzei* [19]. We retrospectively tested some of the specimens collected by Serpa et al. (2021) [19] through real-time PCR, resulting in no amplification of borrelial DNA (data not shown). Although neither of the two *Borrelia*-infected *O. mattogrossae* of the present study were infested by ticks when captured (data not shown), it is widely known that Bbsl are primarily associated with ticks of the genus *Ixodes* [1]. Hence, *I. schulzei* should be further targeted as a putative vector of “*Ca*. B. paulista”.

“*Candidatus* B. paulista” is grouped with *B. carolinensis* in all of the phylogenetic trees constructed for chromosome-encoded genes. *Borrelia carolinensis* was formally described in 2011, cultured from an *Ixodes minor* tick and from *Peromyscus* and *Neotoma* rodents collected in South Carolina, the United States [10]. Thus far, *B. carolinensis* has not been reported from outside southeastern United States [10]. Therefore, it is unlikely that the genospecies characterized in this study corresponds to *B. carolinensis* because it infects a different genus of rodent and because ticks with vastly distanced distributions are implied as their vectors. Interestingly, a phylogeny of plasmid-borne *ospC* of “*Ca*. B. paulista” indicates a relatedness with several strains of *B. carolinensis*, *B. bissettiae*, and *B. burgdorferi* s.s. As OspC modulates mammalian immunological response, favoring the onset of bacterial infection, it has been postulated that the genetic variability of this loci would be shaped by the array of hosts that a given *Borrelia* species infects [13]. Considering that “*Ca*. B. paulista” infects a cricetid rodent species, it is not surprising that its *ospC* sequence is genetically related to homologues characterized from *Borrelia* spp. that merge their cycles also with rodents of this family.

First, the molecular detections of *Borrelia* spp. in South America were based on sequences of *flaB*; therefore, phylogenies for this gene include the majority of genotypes characterized for the region currently. Our phylogenetic analysis for this gene is in the line with that of previous studies [18,38] depicting a monophyletic group of South American Bbsl related to *B. bissettiae* and *B. carolinensis* (Figure 2). The closest genotypes of “*Ca*. B. paulista” correspond to clones A, B, and C detected in *Ixodes fuscipes* (reported as *Ixodes pararicinus*) from Uruguay [38,39] and *Borrelia* sp. Pampa from an *I. longiscutatus* in Brazil [18]. Both ticks might use rodents as hosts, at least for nymphs and larvae [40]. Remarkably, further *Borrelia* genotypes detected in South American ticks associated with rodents (i.e., *Ixodes sigelos* and *Ixodes neuquenensis*) are phylogenetically related to *Borrelia chilensis* [14,16]. To date, this evidence demonstrates that at least two main lineages of Bbsl evolved in association with rodents and their ticks in the region.

Finally, “*Ca.* B. paulista” is the third genospecies of Bbsl identified in Brazil [3,18]. As discussed above, the most probable tick host for this novel *Borrelia* sp. is *I. schulzei*, a species not implicated in human parasitism [41]. Therefore, any conjecture of “*Ca.* B. paulista” as a possible human pathogen is still premature and needs further research.

## Figures and Tables

**Table 1:** Distribution of 382 small mammal specimens, organized by order, family, and species, captured in six areas of the state of São Paulo, one area of the Mato Grosso do Sul state, and one area from Mato Grosso state from 2015 to 2018.

Species	Areas								Total (Tissue Samples)
	A1	A2	A3	A4	A5	A6	A7	A8	
Order Didelmorphia									
* Didelphis albiventris*	9	14	2	6	7	-	1	-	39
* Didelphis aurita*	1	-	-	-	-	2	-	-	3
* Gracilinanus agilis*	-	-	5	17	7	-	28	2	59
* Gracilinanus microtarsus*	-	1	2	-	-	-	-	4	7
* Marmosa* (*Micoureus*) *constantinae*	-	-	-	2	-	2	-	-	4
* Monodelphis domestica*	-	-	-	-	-	-	-	11	11
* Phylander* sp.	-	-	-	1	-	-	-	-	1
Order Rodentia									
* Rattus rattus*	1	14	-	-	10	-	-	-	25
* Mus musculus*	-	1	-	-	-	-	-	-	1
* Oecomys aff. marmorae*	-	-	-	1	-	-	11	13	25
* Nectomys squamipes*	-	-	-	4	10	-	-	-	14
* Necromys lasiurus*	-	-	9	1	-	-	4	-	14
* Oligoryzomys* spp.	8	1	16	24	20	-	-	-	69
* Juliomys* cf. *ossitenuis*	-	-	-	2	-	-	-	-	2
* Akodon* sp.	-	-	-	5	2	6	-	-	13
* Hylaeamys megacephalus*	-	-	-	3	1	-	30	-	34
* Euryoryzomys russatus*	-	-	3	-	-	9	-	-	12
* Cavia* sp.	-	-	2	1	-	-	-	-	3
* Clyomis laticeps*	-	-	-	-	-	-	-	1	1
* Thrichomys pachyurus*	-	-	-	-	-	-	-	6	6
* Cerradomys* sp.	-	-	-	1	-	-	-	-	1
* Cerradomys subflavus*	-	-	-	-	-	-	1	-	1
* Dasyprocta azarae*	-	-	-	-	-	-	4	-	4
* Oecomys* sp. 1	-	-	-	-	-	-	8	-	8
* Oecomys* sp. 2	-	-	-	-	-	-	2	-	2
Not identified	-	-	3	1	-	-	16	-	20
Order Cingulata									
* Dasypus novemcinctus*	1	-	-	-	-	-	-	-	1
Order Carnivora									
* Nasua nasua*	1	-	-	-	-	-	-	1	2
**Total**	21	31	42	69	57	19	105	38	382
